# Multiple Retransplantations After BK Polyomavirus–Associated Metastatic Collecting Duct Carcinoma: Evidence for Protective BK-specific T-Cell Immunity

**DOI:** 10.1097/TP.0000000000005859

**Published:** 2026-09-22

**Authors:** Nathalie Hammer, Nina Khanna, Fadi Haidar, Hans H. Hirsch

**Affiliations:** 1 Division of Nephrology and Hypertension, Department of Medicine, Geneva University Hospital, Geneva, Switzerland.; 2 Division of Infectious Diseases, University Hospital Basel, Basel, Switzerland.; 3 Department of Biomedicine, University of Basel, Basel, Switzerland.; 4 Translational Virology Immunity Pathology, Immunology Research, Department of Medical Biology, UiT The Arctic University of Norway, Tromsø, Norway.

BK polyomavirus (BKPyV)–associated malignancies are rare but devastating complications of transplantation. In 2021, Meier et al. reported on immunologic clearance of donor-derived metastatic BKPyV-associated collecting duct carcinoma following kidney- and pancreas-transplantectomy, metastasectomy, withdrawal of immunosuppression, and interleukin-2 therapy.^1^ While this landmark report emphasized the potential of immune-mediated cancer remission, retransplantation after BKPyV-nephropathy and metastatic BKPyV-associated cancer have not been attempted.

Here, we report the clinical and immunologic follow-up of this patient more than a decade after the initial cancer diagnosis. The detailed clinical course and oncologic management have been described previously.^1^ Briefly, after 8 years of cancer-free survival, the patient underwent kidney retransplantation followed by 2 pancreatic islet infusions under contemporary immunosuppression (Figure 1**, left panel**).

**Figure 1.**
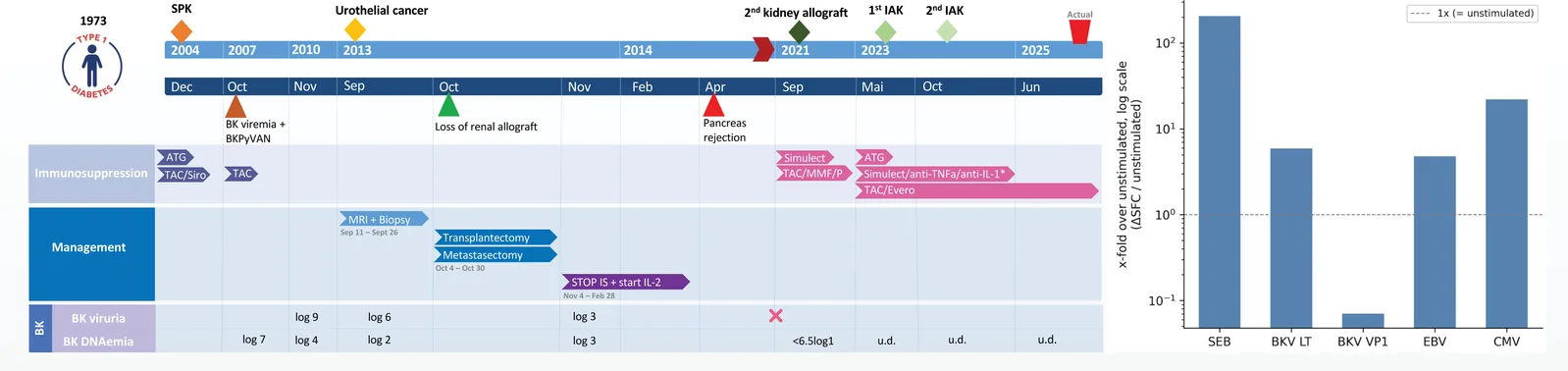
Transplantation history, clinical management and virological results Left panel: Timeline overview; right panel: BKPyV-specific cellular immune responses Abbreviations: anti-lL-1, anti-interleukin 1; anti-TNFa, anti-tumor necrosis factor alpha; ATG, thymoglobulines; BK, BK Polyomavirus; BKPyVAN, BK-associated nephropathy; Evero, everolimus; IAK, islet after kidney transplantation; IL-2, interleukine-2; IS, immunosuppression; MMF, mycophenolate mofetil; MRI, magnetic resonance imagery; P, prednisone; Siro, sirolimus; SPK, simultaneous pancreas-kidney transplantation; TAC, tacrolimus . BK viruria is expressed in log10 (copy/ml); BK DNAemia is expressed in log10 (copy/ml until 2022, then in IU/ml). u.d. undetectable. *Induction immunosuppression according to Edmonton protocol for both IAK. BKPyV-LTag covers the viral tumor antigen 15mer peptides, whereas BKPyV-Vp1 15mer peptides covers the capsid antigen. IFN-γ response expressed as ΔSFC (spot forming cells per million PBMCs, ΔSFC/10^6^ cells). ΔSFC represents the net BKPyV-specific response after background substraction, accounting for baseline T-cell activation and nonspecific IFN-γ secretion observed in unstimulated conditions. Unstimulated wells reflect baseline T cell activity irrespective of antigen specificity. Assay controls were adequate, with a negative control of 23.3 SFC/10^6^ PBMCs and a strong response to Staphylococcal Enterotoxin B (SEB; Δ 4,887 SFC/10^6^ PBMCs). SEB served as a positive control for nonspecific T cell activation. EBV was assessed using consensus 15mer peptide pool and CMV using pp65 15mer peptide pools.

At the most recent evaluation, 4 years after kidney retransplantation and 2 years after the second of two islet infusions, kidney allograft function remained stable, with no detectable donor-specific antibodies. Glycaemic control was adequate under basal insulin (7 UI/day). Despite continued immunosuppression, there was no evidence of recurrent BKPyV replication or malignancy.

To investigate whether durable antiviral immunity contributed to this favorable outcome, BKPyV-specific cellular immune responses were assessed using IFN-γ ELISpot. A strong response against BKPyV large T antigen (LTag) was detected (Δ140 spot-forming cells/10^6^ PBMCs), whereas virtually no response against the capsid antigen Vp1 was observed (Δ2 spot-forming cells/10^6^ PBMCs). However, robust responses against cytomegalovirus and Epstein–Barr virus confirmed preserved virus-specific cellular immunity (Figure 1**, right panel**).

These findings are notable for several reasons. First, they demonstrate that successful kidney and islet retransplantation can be achieved after complete remission of metastatic BKPyV-associated collecting duct carcinoma. Such cases are exceptionally rare, and significant concerns regarding recurrent BKPyV replication and/or tumor relapse have precluded retransplantation in this setting. However, current international guidelines acknowledge that retransplantation after BKPyV-associated graft loss is feasible once viral replication has been immunologically controlled, although experience after BKPyV-associated malignancy is lacking.^2^

Second, the immunologic profile suggests potential mechanisms underlying sustained protection. Cellular immunity is a major determinant of BKPyV control, and BKPyV-specific CD8^+^ T-cell responses directed against the LTag have been associated with declining BKPyV-DNAemia and viral clearance.^3^ Notably, persistent LTag-specific cellular immunity in our patient, the absence of BKPyV-DNAemia, and sustained cancer remission, suggests durable antiviral immune control despite repeated transplantation procedures and ongoing immunosuppression.

LTag is of particular interest because it is essential for viral replication and is implicated in BKPyV-mediated oncogenesis. In contrast to capsid proteins such as Vp1, LTag may remain expressed in transformed cells even when productive viral replication is absent.^4^ We note that re-transplantation of a DBD-kidney involved HLA-matching of 5/6 including HLA-B8 known to present immunodominant LTag-9mer peptides.^3^ LTag-derived epitopes presented by common HLA class I alleles can trigger cytotoxic CD8^+^ T-cell responses, which may also be relevant for adoptive T cell therapy.^5^ Consequently, LTag-specific T-cell responses may protect not only from replicative but also from oncogenic BKPyV-events.

A potential limitation of this report is the absence of longitudinal immune monitoring from the time of cancer remission through retransplantation. Consequently, the dynamics of BKPyV-specific immunity remain undefined. Nevertheless, the current findings, with the concomitant preservation of CMV- and EBV-specific T-cell responses, support intact virus-specific cellular immunity, and provide a unique snapshot of durable immune control in an otherwise unprecedented clinical scenario.

In conclusion, this long-term follow-up of a previously reported case^1^ demonstrates successful kidney retransplantation and subsequent pancreatic islet transplantation after complete remission of metastatic BKPyV-associated collecting duct carcinoma. BKPyV LTag-specific cellular immunity, together with sustained absence of BKPyV-DNAemia and cancer recurrence, supports the hypothesis that antigen-specific immune monitoring may help refine risk assessment and retransplantation strategies in selected patients with prior severe BKPyV-associated disease.

## References

[1] Meier RPH Muller YD Dietrich PY. Immunologic clearance of a BK Virus-associated metastatic renal Allograft Carcinoma. Transplantation. 2021;105:423–429. 10.1097/TP.0000000000003193 PMC7837753 32091486

[2] Kotton CN Kamar N Wojciechowski D.; Transplantation Society International BK Polyomavirus Consensus Group. The second international consensus guidelines on the management of BK polyomavirus in kidney transplantation. Transplantation. 2024;108:1834–1866. 10.1097/TP.0000000000004976 PMC11335089 38605438

[3] Leboeuf C Wilk S Achermann R.; Swiss Transplant Cohort Study. BK polyomavirus-specific 9mer CD8 T cell responses correlate with clearance of BK viremia in kidney transplant recipients: first report from the swiss transplant cohort study. Am J Transplant. 2017;17:2591–2600. 10.1111/ajt.14282 28326672

[4] Müller DC Rämö M Naegele K. Donor-derived, metastatic urothelial cancer after kidney transplantation associated with a potentially oncogenic BK polyomavirus. J Pathol. 2018;244:265–270. 10.1002/path.5012 29205775

[5] Wilhelm M Geng A Schaub S. Posoleucel for BK polyomavirus in kidney transplant recipients. J Am Soc Nephrol. 2024;35:1784–1785. 10.1681/ASN.0000000000000500 PMC11617477 39302720

